# Help-Seekers in an Early Detection of Psychosis Service: The Non-cases

**DOI:** 10.3389/fpsyt.2021.778785

**Published:** 2021-12-10

**Authors:** Line Lindhardt, Morten Lindhardt, Ulrik Helt Haahr, Lene Halling Hastrup, Erik Simonsen, Julie Nordgaard

**Affiliations:** ^1^Early Psychosis Intervention Centre, Mental Health Services East, Psychiatry Region Zealand, Roskilde, Denmark; ^2^Department of Occupational and Social Medicine, Holbæk Hospital, Holbæk, Denmark; ^3^Department of Internal Medicine, Holbæk Hospital, Holbæk, Denmark; ^4^Psychiatric Research Unit, Psychiatry Region Zealand, Slagelse, Denmark; ^5^Danish Centre for Health Economics, University of Southern Denmark, Odense, Denmark; ^6^Department of Clinical Medicine, Faculty of Health and Medical Sciences, University of Copenhagen, Copenhagen, Denmark; ^7^Mental Health Centre Amager, Copenhagen, Denmark

**Keywords:** diagnoses, help-seekers, early intervention, early detection, clinical diagnostics, psychosis, schizophrenia, first episode

## Abstract

**Purpose:** Enhancing early help-seeking is important for early intervention in psychosis. However, knowledge is limited about those help-seekers who are not initially found to have psychotic symptoms when assessed in services aiming at psychosis detection and, thus, deemed ineligible for early intervention of psychosis programs. We aimed to examine clinical diagnostic and socioeconomic pathways of help-seekers accessing an early detection of psychosis service with referral-free access. Specific focus was on the help-seekers initially assessed not to have psychotic symptoms, considered the non-cases, and to examine potential differences and similarities between non-cases and cases (i.e., those initially assessed to have psychotic symptoms).

**Methods:** We followed 450 help-seekers assessed by a free-of-referral early detection of psychosis team in national registers for up to 4 years. We examined clinical diagnoses and status of not in education, employment, or training (NEET) before and after contact with the team.

**Results:** Of the non-cases, 46% were referred to mental health services by the early detection of psychosis team for evaluation of other mental disorders, and 15% of these were subsequently diagnosed with a non-affective psychotic disorder during follow-up of 12–52 months. Prior to current help-seeking, 39% (*n* = 174) of the help-seekers had had contact with other mental health services. Nearly a quarter of help-seekers were NEETs at the time of assessment; the number increased during follow-up, both for cases and non-cases. Of the cases, 58% were subsequently clinically diagnosed by mental health services. Those seeking help who had no previous contact with mental health services were more frequently diagnosed with a non-affective psychotic disorder during follow-up (*p* = 0.05).

**Conclusion:** Referral-free services to promote early detection of psychosis seem a valuable add-on to established pathways, allowing early intervention in psychosis. Our results point to an unmet mental health service need among non-cases; overall, in our sample, independent of case status, social functioning was markedly affected. Our results have implications for future focus in early detection of psychosis. Offering intervention to non-cases within the service has the potential to be cost effective, e.g., if a timely and targeted intervention reduces repeated contacts in other mental health services and social services.

## Introduction

Programs that intervene early in psychosis are widely acknowledged to improve treatment response and give greater patient satisfaction with treatment (1). Nevertheless, there is substantial delay in establishing contact with mental health services after the psychotic symptoms emerge (2–4). Several factors contributing to this delay have been identified, for example, not identifying the problems as psychiatric in nature, hoping the problems will resolve by themselves, and embarrassment and fear of stigmatization (5–7). Further complicating early help-seeking is the lack of acknowledgment of the problems, which is an inherent feature of psychosis (8).

Services aiming at early detection of untreated psychosis have been introduced in an attempt to enhance the help-seeking process (4). The services are low threshold, can be accessed easily, and deliver immediate clinical attention and evaluation of psychotic symptoms (9). Different initiatives have been launched through the services in an attempt to promote help-seeking behavior, for example, raising awareness in the general population of the signs and symptoms of psychosis; educating professionals who have contact with adolescents and young adults, such as teachers and social service workers, to recognize psychotic symptoms; and reducing barriers to accessing treatment (4, 10). Societal disconnection among young adults accessing mental health services has been suggested as a risk marker in the trajectory of mental illness (11). Young adults experiencing a first-episode psychosis are often socially disconnected at the time of service access (12, 13), and delay in accessing early intervention has been found among first-episode psychosis individuals being not in education, employment, or training (NEET) (14).

Enhancing help-seeking behavior has the potential to increase early contacts to the specialized intervention services, leading to better outcomes (15–17). However, ethical dilemmas are involved, and to date, these have not been adequately addressed. For example, could an increase in help-seeking lead to healthy young people being pathologized? What happens to the non-cases, that is, the young people who experience difficulties and who often have a low level of social functioning but who are not found psychotic and are, thus, not offered treatment in the specialized intervention of psychosis teams (18)?

In this study, we aimed at examining the clinical diagnostic and socioeconomic pathways of a help-seeking population accessing a free-of-referral service for early detection of psychosis. Specific focus was on the help-seekers not found with psychotic symptoms at the evaluation, here considered the non-cases, and to examine potential differences and similarities between non-cases and those evaluated to have psychotic symptoms, considered the cases.

## Methods

### Setting

#### The Early Detection Team

In addition to the nationally implemented early intervention of psychosis program, one region in Denmark has implemented an outreach early detection team TOP (in Danish: TOP, tidlig opsporing af psykose). The service is targeted toward the general population and has free-of-referral access, whereas the early detection team functions alongside established referral pathways, e.g., from GPs or internally after mental hospital admissions, and provides direct access to evaluation in specialized mental health services. In Denmark, the public mental health service is free of charge, and the main part of the diagnostic evaluation and treatment of psychosis is placed in the public service.

The early detection team is located in Roskilde, Denmark. The catchment area is one of five regional health authorities of Denmark, Region Zealand, and is primarily rural or suburban. The region has 834,000 inhabitants.

The team can be contacted directly by all residents of the region, including their relatives, general practitioners, and others in contact with individuals whose symptoms might cause concern regarding psychosis. Telephone screening is used to determine whether the caller is in the target group of the team; if this is the case, the individual is then offered an in-person assessment within one working day. The early detection of psychosis team can be contacted anonymously, but identification is required for face-to-face assessment, which can take place in the neighborhood of the interviewee.

An information campaign was launched in 2012 to raise awareness in the community of the early-detection team and to increase knowledge about early signs of psychosis among the population in the catchment area.

The eligibility criteria for assessment by the early detection team are help-seekers for whom telephone screening have raised the slightest suspicion of symptoms that could cause concern of psychotic symptoms, age between 15 and 65 years, not previously treated in mental health outpatient services for a non-affective psychotic disorder, and those who at the time of contact are not undergoing treatment for any mental disorder in outpatient services.

#### Assessment by the Team

Nurses with special training in psychopathology conduct face-to-face interviews. If during the subsequent assessment the nurse assesses the help-seeker to have psychotic symptoms, a referral is made for a clinical diagnostic evaluation in the local outpatient mental health service. Thus, the assessment by the team is based on a single in-person contact. In case of the assessment revealing psychotic symptoms, the help-seeker is referred into mental health services and diagnostic evaluation is initiated here within a fortnight.

If the assessment does not raise suspicion of a psychotic disorder, but raises concern of a mental disorder other than a non-affective psychotic disorder, a referral is made to the mental health service section of relevance for diagnostic evaluation.

The assessments take into account sociodemographic information, psychosocial history, and a comprehensive assessment of psychopathology using, among others, the Positive and Negative Symptoms Scale (PANSS) (19). A senior psychiatrist or psychologist supervises evaluation of all interviews conducted and referral to further diagnostic evaluation and treatment in psychiatric services is decided in agreement within the team.

#### Study Population

Data consisted of administrative data from the early detection team. The study population consisted of help-seekers who were assessed by the early detection team, from February 2012 to June 2015 and who disclosed their personal identification number.

Assessments conducted by the early detection team in the inclusion period had been supervised by a senior psychiatrist with substantial research experience (UHH or JN) (20–23).

### Measures

#### Data From the Early Detection Team Assessment

We used systematically collected administrative data based on the registration after the assessment by the clinician in the early detection team. The registrations included sociodemographic information, the source of referral to the team, tentative diagnosis allocated by the team, and information about referrals to mental health services. Individuals were identified *via* the unique personal identification number through which all citizens in Denmark can be identified (24).

Help-seekers who were assessed as having psychotic symptoms and who were referred to diagnostic evaluation of a non-affective psychotic disorder were considered as cases. Help-seekers who were assessed not to have psychotic symptoms at assessment in the early detection of the psychosis team were considered as the non-cases.

#### Register Data of Clinical Diagnoses

Data from The Danish Psychiatric Central Research Register (DPCR) (25) were obtained for each person by linkage to the national register *via* the personal identification number using an encrypted identification key. The register contains information on medical diagnoses classified according to the International Classification of Diseases, Tenth Revision (ICD-10) (26) registered for all inpatient admissions and for all outpatient contacts since January 1995.

Diagnoses were categorized according to the ICD-10 codes as non-affective psychotic disorder with medical codes DF20-29 and other mental disorder with medical codes DF00-19 and DF30-99. Schizophrenia with ICD-10 code DF20 was sub-grouped within the non-affective psychotic disorders. The non-affective disorder category included schizotypal disorder with the medical code DF21.

Prior mental disorder was measured by obtaining data from the DPCR from 1995 and until assessment in the early detection team. Prior mental disorder was categorized as non-affective psychotic disorder, other mental disorder, and no prior mental disorder.

Mental disorder in follow-up was measured by obtaining data from the DPCR from the time of assessment in the early detection team until June 2016 and categorized equivalent to the measure of prior mental disorder.

#### Register Data of Social Transfer Income

Data from the DREAM database of The Ministry of Employment, a national database collecting data since 1991 on all social transfer income (27), were obtained encrypted *via* the personal identification number to provide a measure of NEET (28). The database covers the entire population of Denmark. The data are updated weekly and are suitable for analyzing changes in employment status. Database codes registered for each person were obtained on social transfer income of non-medical benefits, medical benefits, and disability pension. The data were derived from the register equaling the week of assessment in the early detection service, i.e., baseline, and subsequently data were derived from the register equaling the same week 1 year preceding the assessment and the same week in the subsequent year after assessment. The measure of NEET was defined as present if a code of social transfer income was registered in the DREAM database and absent if there was no registration.

The measure was divided into NEET status at the time of assessment, 1 year before assessment and 1 year after assessment.

### Ethics and Approvals

The study was approved by the Danish Data Protection Agency, journal no. Reg-138-2015.

### Statistical Analysis

Initially, a descriptive analysis of the characteristics of the study population was made.

To identify associations between the interview assessment in the early detection team and a later clinical diagnosis of non-affective psychotic disorder, we initially applied a descriptive analysis by subdividing the non-cases in those referred in mental health services by the early detection team for diagnostic evaluation of other non-psychotic disorders and those non-cases not found eligible of referral into mental health services by the early detection of psychosis team. We used chi-square to test for differences between the groups of non-cases referred with symptoms of other mental disorders, non-cases not eligible for referral in mental health services, and the cases.

We tested for associations of the study population characteristics (case status, mental health service use preceding assessment, NEET-status, age, and sex) with a later clinical diagnosis of a non-affective psychotic disorder in follow-up with use of chi-square or *t*-test, depending on the type of variable.

Additionally, we divided all the individuals who were found to have a psychotic disorder during follow-up into two groups: group 1 for those who were also found to have psychosis by the early detection team (cases), and group 2 for those who were not found to suffer from psychosis by the early detection team (non-cases) but who were later diagnosed with psychosis. Next, we tested for potential differences between the 2 groups in relation to their use of mental health services prior to assessment, NEET-status, age, and sex. Chi-square or *t*-test were used depending on the variable applied.

We developed a Cox regression model to test for time-dependent differences in the outcome of a clinical diagnosis of non-affective psychotic disorder between the cases and non-cases as being the reference group. The non-cases were subdivided further into two groups of either non-cases referred with symptoms of other mental disorder than non-affective psychotic disorder, or non-cases not eligible for referral in mental health services. The assumptions for Cox regression model were met with proportionality in risk for each group in the model. The model was censored for previously diagnosed non-affective psychotic disorder and evaluated a psychotic disorder-free follow-up, in cases vs. non-cases referred with symptoms of other mental disorder vs. non-cases not found eligible for referral.

All statistical analyses were conducted using SAS (SAS Institute, CA & Cary, NC, USA; Enterprise Guide version 7.11/SAS 9.4). Microsoft Excel was used to create the graphical plots.

## Results

### Assessment and Referral

**Figure 1:**
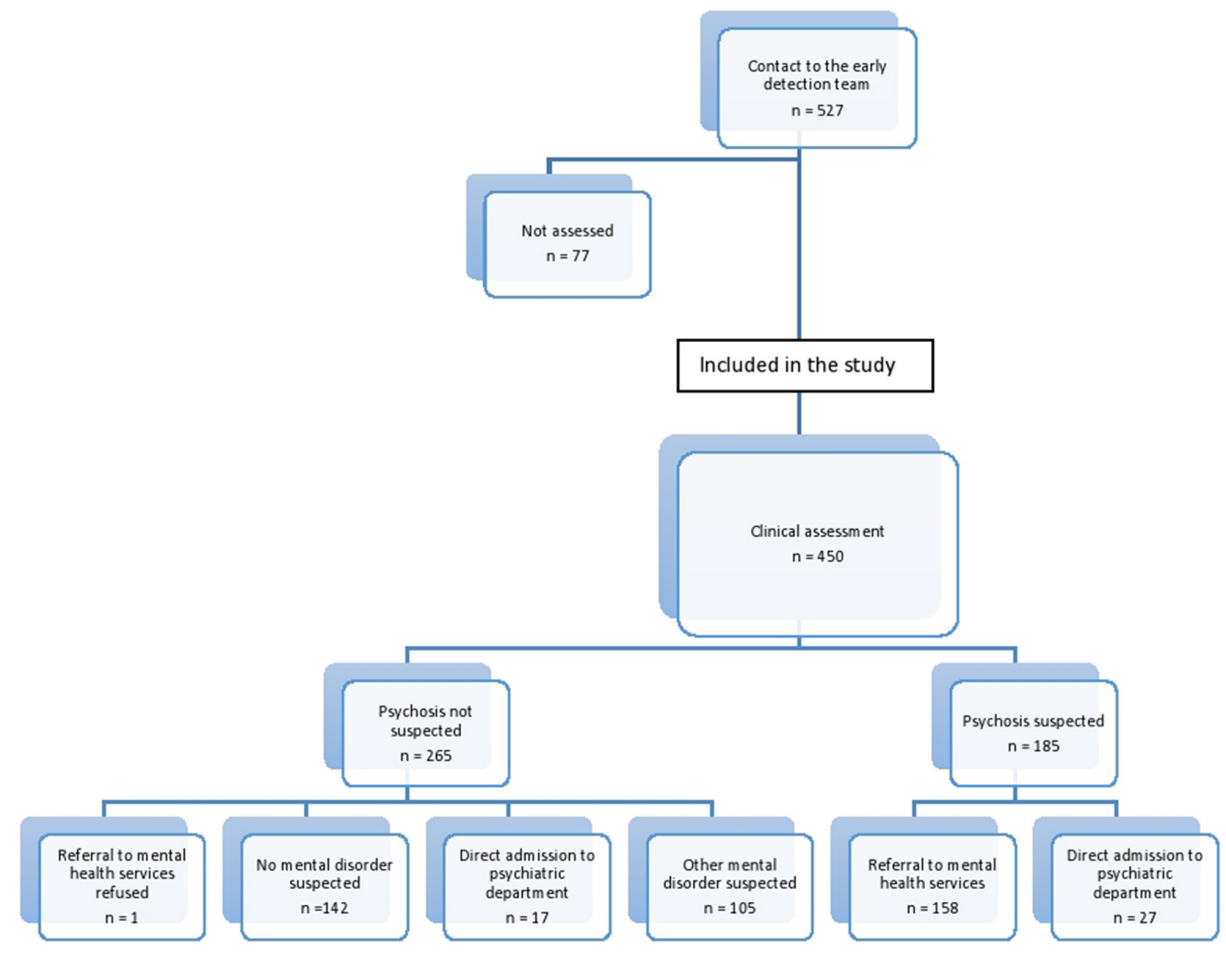
Flow-chart of the study population, assessment by the early detection team and referrals.

The early detection of psychosis team established contact with 527 individuals, of whom 450 fulfilled the inclusion criteria. Figure 1 gives an overview.

Of the help-seekers considered as non-cases, i.e., not presenting with psychotic symptoms at assessment in the early detection of psychosis team and therefore not referred for diagnostic evaluation for non-affective psychotic disorder following assessment, 123 (46 %) were found eligible for referral to mental health services for evaluation of other mental disorders. One person refused referral.

### Social Service Contact

Contact to the early detection team was initiated by parents or by help-seekers themselves in the majority of cases. Contact to the team was initiated by caseworkers in the social services in 10%. Of help-seekers, 24% were NEET at the time of assessment, with no difference between groups of cases and non-cases (*p* = 0.35). The prevalence increased from 1 year before assessment to 1 year after assessment, as shown in Table 1.

**Table 1:** Study population characteristics (*n* = 450 help-seekers).

	***N* (%) or mean (SD)**
**Sex (%)**	
Males	264 (58.7)
Females	186 (41.3)
**Age, mean (years) (SD)**	20.6 (7.2)
Ethnicity among welfare benefit recipients^*^ **(%)**	
Danish ethnicity	157 (93.5)
Migrants	5 (3.0)
Migrant descendants	4 (2.4)
**Marital status (%)**	
Unmarried	429 (97.5)
Married	4 (0.9)
Divorced/widow	7 (1.6)
**Household status (%)**	
Living alone	156 (35.5)
Living in a family	164 (37.2)
Household with more families	120 (27.3)
**Not in education, employment, or training (NEET)-status (receiving non-medical and medical welfare benefits) (%)**	
1-year preceding assessment	57 (12.7)
At time of help-seeking in early detection team	110 (24.4)
1-year after assessment	132 (29.3)
**Initiative to referral to early detection team (%)**	
Self	103 (22.9)
Parents	104 (23.1)
GP	100 (22.2)
Social services	44 (9.8)
Other	99 (22.0)
**Case status by assessment (%)**	
Cases	185 (41.1)
Non-cases	265 (58.9)
**Prior diagnosed mental disorder (%)**	
Diagnosed other mental disorder	146 (32.4)
Diagnosed psychotic disorder	28 (6.2)
No prior mental disorder	276 (61.3)

* *Among welfare benefit recipients (n = 168)*.

### Mental Disorders Prior to Assessment

With a mean age of 21.5 years (SD 6.1) at the time of assessment by the early detection team, 174 help-seekers (39%) had a prior mental disorder, and 28 (6%) had prior non-affective psychotic disorder (see Table 1). In help-seekers with prior non-affective psychotic disorder, the median time since diagnosis was 1.4 years (IQR 0.95–3.30).

Of the 61% with no prior mental disorder, 115 (42%) were found to be cases of first-episode psychosis, and 161 (58%) were considered non-cases.

### Mental Disorders in Follow-Up

During follow-up, 317 (70%) help-seekers were subsequently clinically diagnosed by the mental health services, with a maximum follow-up of 4 years and 4 months, and a minimum follow-up of 12 months on an individual level. The majority of those diagnosed were males (59 vs. 41%, *p* < 0.01). Nearly one-third (*n* = 146; 32%) of the help-seekers were diagnosed with a non-affective psychotic disorder in follow-up and half of them with schizophrenia (*n* = 73; 16%).

Help-seekers considered as cases were subsequently diagnosed with a non-affective psychotic disorder during the follow-up in 58% of cases. Of the non-cases, 154 (58%) were diagnosed with a mental disorder in follow-up and 39 (15%) were in follow-up diagnosed with a non-affective psychotic disorder. Among the non-cases referred with symptoms of other mental disorders, 19% had non-affective psychotic disorder in follow-up, and among the non-cases found not eligible for referral, 11% had non-affective psychotic disorder in follow-up. There was significant difference between cases and non-cases in non-affective psychotic disorder in follow-up (see Table 2).

**Table 2:** Mental disorders during follow-up.

	**Non-affective psychotic disorder in follow-up**	**Other mental disorder in follow-up**	**Schizophrenia^†^ in follow-up**
	***n* (%)**	***n* (%)**	***n* (%)**
Non-cases not eligible for referral in mental health services (*n* = 142)^#^	16 (11.2)	46 (32.4)	9 (6.3)
Non-cases referred with symptoms of other mental disorder (*n* = 122)^#^	23 (18.9)	69 (56.6)	8 (6.6)
Cases of non-affective psychosis (*n* = 185)	107 (57.8)^*^	55 (29.7)	56 (30.3)^*^
All assessed (*n* = 450)	146 (32.4)	171 (38.0)	73 (16.2)

# *Non-cases are divided into non-cases not eligible for referral in mental health services and non-cases referred with symptoms of other mental disorder than psychosis*.

# *Difference between cases and non-cases, chi-square-test (p < 0.01)*.

† *Subgroup of non-affective pcychotic disorder*.

NEET status was seen in 29% of help-seekers 1 year after the assessment, and there was significant difference between help-seekers with non-affective psychotic disorder in follow-up and help-seekers with no psychotic disorder in follow-up (37.0 vs. 25.7%, *p* = 0.01). NEET status at the time of assessment, NEET status 1 year before the assessment, mean age, and sex did not significantly differ between help-seekers with a non-affective psychotic disorder in follow-up, and those with no psychotic disorder in follow-up, (see Table 3).

**Table 3:** Socio-demographic and clinical characteristics of help-seekers diagnosed and not diagnosed with non-affective psychotic disorder in follow-up.

	**Non-affective psychotic disorder in follow-up**	**No non-affective psychotic disorder in follow-up**	***p* ^†^**
	***n* = 146**	***n* = 304**	
**Assessment by the early detection team (%)**			
Cases	107 (73.3)	78 (25,7)	<0.01^*^
Non-cases	39 (26.7)	226 (74.3)	
**Mental health service use preceding assessment (%)**			
No prior mental disorder	99 (67.8)	177 (58.2)	0.05^**^
Prior mental disorder	47 (32.2)	127 (41.8)	
**NEET status**		
1-year perceiving assessment	18 (12.3)	39 (12.8)	*0.88*
At time of help-seeking in early detection team	39 (26.7)	71 (23.3)	*0.44*
1-year following assessment	54 (37.0)	78 (25.7)	*0.01^#^*
**Mean age (years)**	20.0 (3.8)	20.8 (6.9)	*0.16*
**Sex**			
Male	88 (60.3)	176 (57.9)	*0.63*
Female	58 (39.7)	128 (40.1)	

* *Difference between help-seekers assessed with psychotic symptoms and help-seekers not assessed with psychotic symptoms*.

** *Difference between help-seekers with prior mental disorder and help-seekers with no prior mental disorder*.

† *p-values originates from chi-square-test*.

# *Difference between NEET status and self-support 1 year following assessment*.

The help-seekers with non-affective psychotic disorder in follow-up was more frequently first-time help-seekers than those help-seekers with no psychotic disorder in follow-up (67.8 vs. 58.2%, *p* = 0.05). We found no significant difference between cases and non-cases in mental health service use preceding assessment, NEET-status, age, or sex among help-seekers clinically diagnosed with a non-affective psychotic disorder in follow-up (data not shown).

**Figure 2:**
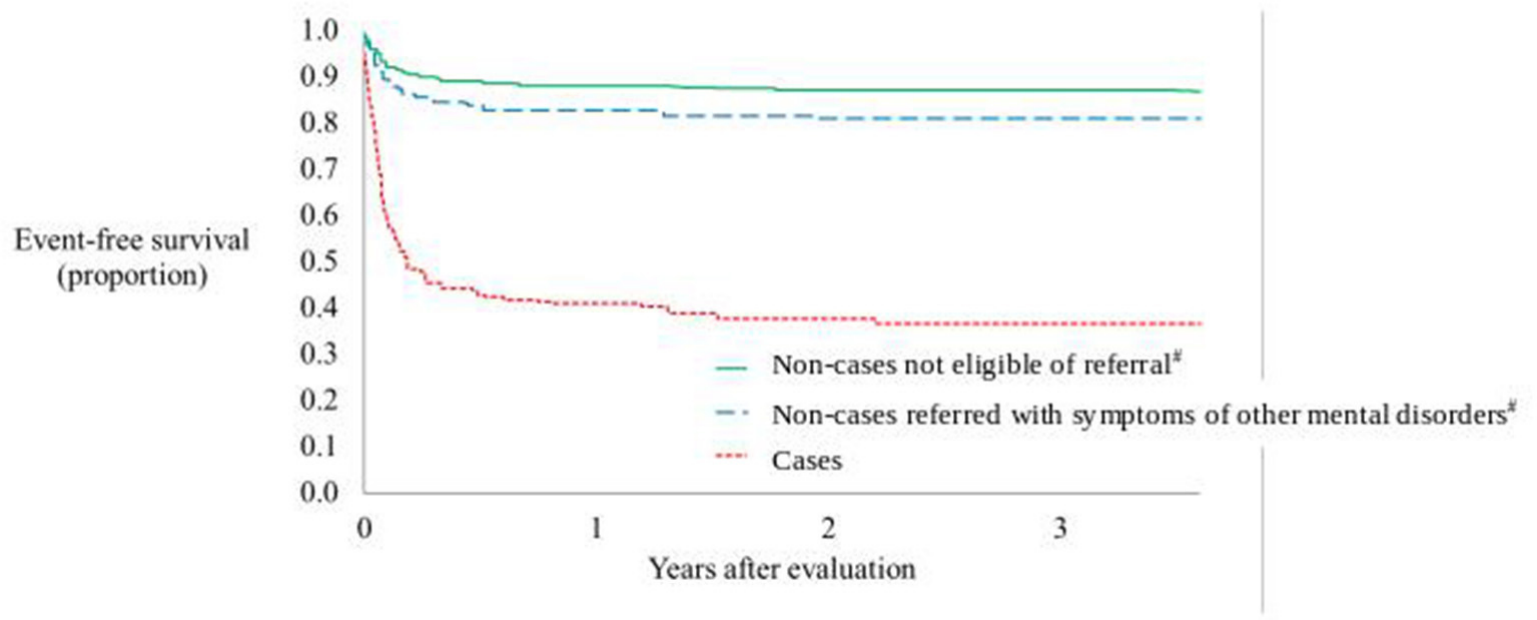
Non-affective psychotic disorder-free period after assessment by the early detection team. ^#^Non-cases are divided into non-cases not eliglible for refferal in mental health services and non-cases referred with symptoms of other mental disorder than psychosis.

For cases, the hazard ratio of non-affective psychotic disorder in follow-up was 4.7 (95% CI 3.0 to 7.4, *p* < 0.0001) compared with non-cases referred with symptoms of other mental disorders. The hazard ratio for cases, compared with non-cases not eligible of referral, was 7.1 (95% CI 4.7–10.9, *p* < 0.0001). We found no difference in the relative event rate of non-affective psychotic disorder in follow-up between non-cases referred with symptoms of other mental disorders and non-cases not eligible of referral (*p* = 0.15). The majority of events of non-affective psychotic disorder in follow-up occurred in the first months after assessment, as shown in Figure 2, with non-affective psychotic disorder-free period after assessment by the early detection team.

## Discussion

This study is the first to examine the diagnostic trajectories and the socioeconomic position of help-seekers evaluated to be cases and those not evaluated to be cases in a service established for early detection of psychosis. The results showed that a substantial part of the non-cases became cases, i.e., those individuals who were assessed not to have psychotic symptoms at the initial assessment were subsequently diagnosed with a non-affective psychotic disorder during the follow-up of a maximum of 4 years and 4 months and a minimum of 12 months on individual level. Moreover, the prevalence of NEET status was similar for non-cases and cases. The help-seekers identified with psychotic symptoms (i.e., cases) by the early detection of psychosis team were subsequently clinically diagnosed with a non-affective psychotic disorder in the majority of cases. Half of these individuals were diagnosed with schizophrenia.

Overall, 59% of assessed help-seekers in the early detection of psychosis service were found to be non-cases, which is similar to findings by Jordan et al. with a non-case rate of 66% in an early intervention service with a supporting information campaign to promote help-seeking (17). However, the non-case rate was somewhat higher than findings by O'Donoghue et al. who reported a case rate of 53% (15). The self-referral option in our study could explain the higher non-case rate compared with O'Donoghue et al.; when targeting a more unselected population, a higher non-case rate will be expected. Nevertheless, applying the self-referral option has the potential to reduce pathway to care by easing access to care (29).

We found that a considerable proportion of non-cases required referral to mental health services because of symptoms of other mental disorders. Moreover, a significant part of the referred non-cases received a clinical diagnosis of non-affective psychotic disorder during follow-up. The latter finding is in corroboration with findings by Edwards et al. (30), with indication of unmet and ongoing mental health needs in the non-case group.

A possible explanation on why some help-seekers were identified as non-cases at the assessment and were then clinically diagnosed with non-affective psychotic disorder during the follow-up could be that the help-seekers were in a prodromal state at the time of contact with the early detection service (31). It could also reflect that some individuals require more time to feel sufficiently secure to disclose their symptomatology or to view the experiences as abnormal.

The similarity in socioeconomic position between help-seekers found with psychotic symptoms and those not found with psychotic symptoms at service access correspond to the results found by Ferarra et al. when comparing individuals with psychosis with those in a prodromal state (32). Thus, it seems that despite not being found with psychotic symptoms initially, the rate of NEET status of the non-cases differs from that of mentally healthy young people (33).

In addition, NEET status among help-seekers were frequent and increased from 1 year before assessment to 1 year after contact to the early detection service. Help-seekers later diagnosed with non-affective psychotic disorder had the highest rate of NEET status, which is comparable to findings in first-episode psychosis populations (12). Accordingly, this could be indicative of a high disease burden in the help-seekers with impaired social and vocational functioning.

Interestingly, 39% of the help-seekers assessed by the early detection of psychosis team had previously been diagnosed with a mental disorder. In a previous work, we found that 71% of first-episode schizophrenia patients had been in contact with mental health services 1 year preceding clinical diagnosis of schizophrenia (16). The considerable proportion of help-seekers who had already been in contact with mental health services before contact with the early detection team could be a result of a delay in recognizing the psychosis within the mental health services. Several studies have addressed the difficulties mental health staff have in identifying psychotic symptoms, e.g., Kvig et al. demonstrated that initial complaints of neurotic symptoms were associated with delayed identification of a psychotic disorder (34); in another study, poor psychosis detection skills among healthcare professionals were found to lead to delays in the treatment of psychosis (6). Contact with other mental health services before the assessment in the early detection time could as well-reflect a prodromal state presentation at the earlier contact. This proposal is in corroboration with the findings in a recent meta-analysis by Fusar-Poli et al. demonstrating attenuated psychotic symptoms lasting for more than 1 year before their presentation at specialized services (35).

Previous mental health contact could seemingly compromise the achievement of easing the pathway to care of the early detection of psychosis service. However, for 67.8% of those diagnosed with a non-affective psychosis in follow-up, the early detection service was the first contact, and thus the direct path to mental health services. This demonstrates the success of the early detection service.

## Strengths and Limitations

A strength of our study was the linking to national longitudinal registers providing follow-up data at individual level as well as information of service use prior to assessment. Retrieving data based on the unique personal identification number secures the capture of interactions with the mental health services.

As for limitations, the use of SCI-PANSS in assessments by the early detection team instead of a full diagnostic instrument is not optimal; this occurred because the service is intended to offer only initial screening for psychotic symptoms. Obviously, this presents the possibility of the tentative diagnosis being wrong; however, we believe that the risk is mostly in the direction of underdiagnosing, not overdiagnosing.

The use of register data catchment of the clinical diagnosis rather than an in-person diagnostic interview at follow-up could be a limitation to interpreting the findings as some help-seekers might have disengaged from intervention in mental health services and, accordingly, not received a clinical diagnosis.

## Conclusion

In conclusion, our findings support the value and importance of early detection services with referral-free access as an add-on to the established pathway in early intervention in psychosis. The findings also point to an unmet mental health service need for non-cases. We did not find any evidence indicating that early detection led to pathologizing of healthy young people, but we did find that a substantial part of help-seekers initially evaluated as non-cases (i.e., no psychotic symptoms) were later diagnosed with a non-affective psychotic disorder. Additionally, the level of social functioning of non-cases was comparable to those initially assessed with psychotic symptoms.

The findings of clinically diagnosed other mental disorders preceding a first-episode psychosis highlight the importance of re-evaluation of the diagnosis in young adults with repeated service needs. Our results have implications for future focus in the early detection of psychosis. Offering intervention to non-cases within the service has the potential to be cost effective, e.g., if a timely and targeted intervention reduces repeated contacts in other mental health services and social services. Future research in interventions and outcomes in non-case groups is needed.

## Data Availability Statement

The original contributions presented in the study are included in the article/supplementary files, further inquiries can be directed to the corresponding authors.

## Ethics Statement

Ethical review and approval was not required for the study on human participants in accordance with the local legislation and institutional requirements. Written informed consent from the participants' legal guardian/next of kin was not required to participate in this study in accordance with the national legislation and the institutional requirements.

## Author Contributions

LL, UH, and JN obtained applicable governmental approvals and carried out the register data collection. LL, JN, LH, and ML performed the data management and statistical analyses. LL wrote the first draft of the manuscript and JN contributed. All authors contributed with valuable input to the critical revision of the manuscript and which has been approved by all authors. All authors participated in the idea-generating process of the study.

## Conflict of Interest

The authors declare that the research was conducted in the absence of any commercial or financial relationships that could be construed as a potential conflict of interest.

## Publisher's Note

All claims expressed in this article are solely those of the authors and do not necessarily represent those of their affiliated organizations, or those of the publisher, the editors and the reviewers. Any product that may be evaluated in this article, or claim that may be made by its manufacturer, is not guaranteed or endorsed by the publisher.

## References

[1] Fusar-Poli P McGorry PD Kane JM. Improving outcomes of first-episode psychosis: an overview. World Psychiatry. (2017) 16:251–65. 10.1002/wps.20446 28941089 PMC5608829

[2] Maric NP Raballo A Rojnic Kuzman M Andric Petrovic S Klosterkotter J Riecher-Rossler A. European status and perspectives on early detection and intervention in at-risk mental state and first episode psychosis: viewpoint from the EPA section for prevention of mental disorders. Eur Psychiatry. (2017) 46:48–50. 10.1016/j.eurpsy.2017.08.003 29017063

[3] Kvig EI Brinchmann B Moe C Nilssen S Larsen TK Sorgaard K. Geographical accessibility and duration of untreated psychosis: distance as a determinant of treatment delay. BMC Psychiatry. (2017) 17:176. 10.1186/s12888-017-1345-8 28486982 PMC5424409

[4] Lloyd-Evans B Crosby M Stockton S Pilling S Hobbs L Hinton M. Initiatives to shorten duration of untreated psychosis: systematic review. Br J Psychiatry. (2011) 198:256–63. 10.1192/bjp.bp.109.075622 21972275

[5] Clement S Schauman O Graham T Maggioni F Evans-Lacko S Bezborodovs N. What is the impact of mental health-related stigma on help-seeking? A systematic review of quantitative and qualitative studies. Psychol Med. (2015) 45:11–27. 10.1017/S0033291714000129 24569086

[6] Bay N Bjornestad J Johannessen JO Larsen TK Joa I. Obstacles to care in first-episode psychosis patients with a long duration of untreated psychosis. Early Interv Psychiatry. (2016) 10:71–6. 10.1111/eip.12152 24861169

[7] Jansen JE Woldike PM Haahr UH Simonsen E. Service user perspectives on the experience of illness and pathway to care in first-episode psychosis: a qualitative study within the TOP project. Psychiatr Q. (2015) 86:83–94. 10.1007/s11126-014-9332-4 25464933

[8] Nordgaard J Nilsson LS Gulstad K Buch-Pedersen M. The paradox of help-seeking behaviour in psychosis. Psychiat Quart. (2021) 92:549–59. 10.1007/s11126-020-09833-3 32821995

[9] Johannessen JO McGlashan TH Larsen TK Horneland M Joa I Mardal S. Early detection strategies for untreated first-episode psychosis. Schizophr Res. (2001) 51:39–46. 10.1016/S0920-9964(01)00237-7 11479064

[10] Joa I Johannessen JO Auestad B Friis S McGlashan T Melle I. The key to reducing duration of untreated first psychosis: information campaigns. Schizophr Bull. (2008) 34:466–72. 10.1093/schbul/sbm095 17905788 PMC2632428

[11] Cross SPM Scott J Hickie IB. Predicting early transition from sub-syndromal presentations to major mental disorders. BJPsych Open. (2017) 3:223–7. 10.1192/bjpo.bp.117.004721 28959452 PMC5596309

[12] Cotton SM Lambert M Schimmelmann BG Filia K Rayner V Hides L. Predictors of functional status at service entry and discharge among young people with first episode psychosis. Soc Psych Psych Epid. (2017) 52:575–85. 10.1007/s00127-017-1358-0 28233045

[13] Addington J Addington D. Patterns of premorbid functioning in first episode psychosis: relationship to 2-year outcome. Acta Psychiatr Scand. (2005) 112:40–6. 10.1111/j.1600-0447.2005.00511.x 15952944

[14] Iyer S Mustafa S Gariepy G Shah J Joober R Lepage M. A NEET distinction: youths not in employment, education or training follow different pathways to illness and care in psychosis. Soc Psychiatry Psychiatr Epidemiol. (2018) 53:1401–11. 10.1007/s00127-018-1565-3 30094632 PMC6267132

[15] O'Donoghue B Lyne J Renwick L Madigan K Kinsella A Clarke M. A descriptive study of 'non-cases' and referral rates to an early intervention for psychosis service. Early Interv Psychiatry. (2012) 6:276–82. 10.1111/j.1751-7893.2011.00328.x 22240056

[16] Hastrup LH Haahr UH Nordgaard J Simonsen E. The effect of implementation of an early detection team: a nationwide register-based study of characteristics and help-seeking behavior in first-episode schizophrenia in Denmark. Schizophr Res. (2018) 201:337–42. 10.1016/j.schres.2018.04.034 29706446

[17] Jordan G Kinkaid M Iyer SN Joober R Goldberg K Malla A. Baby or bathwater? Referrals of “non-cases” in a targeted early identification intervention for psychosis. Soc Psychiatry Psychiatr Epidemiol. (2018) 53:757–61. 10.1007/s00127-018-1502-5 29541798

[18] Kline ER. Commentary: unmet need for mental health services among people screened but not admitted to an early psychosis intervention program. Schizophr Res. (2019) 204:2–3. 10.1016/j.schres.2018.12.049 30609956 PMC6402980

[19] Kay SR Fiszbein A Opler LA. The positive and negative syndrome scale (PANSS) for schizophrenia. Schizophr Bull. (1987) 13:261–76. 10.1093/schbul/13.2.261 3616518

[20] Nordgaard J Buch-Pedersen M Hastrup LH Haahr UH Simonsen E. Measuring psychotic-like experiences in the general population. Psychopathology. (2019) 52:240–7. 10.1159/000502048 31454823

[21] Nordgaard J Revsbech R Saebye D Parnas J. Assessing the diagnostic validity of a structured psychiatric interview in a first-admission hospital sample. World Psychiatry. (2012) 11:181–5. 10.1002/j.2051-5545.2012.tb00128.x 23024678 PMC3449355

[22] Haahr U Simonsen E Rossberg JI Johannessen JO Larsen TK Melle I. Patient satisfaction with treatment in first-episode psychosis. Nord J Psychiatry. (2012) 66:329–35. 10.3109/08039488.2011.644808 22250962

[23] Haahr UH Larsen TK Simonsen E Rund BR Joa I Rossberg JI. Relation between premorbid adjustment, duration of untreated psychosis and close interpersonal trauma in first-episode psychosis. Early Interv Psychiatry. (2018) 12:316–23. 10.1111/eip.12315 26800653

[24] Pedersen C. The Danish civil registration system. Scand J Public Health. (2011) 39:22–5. 10.1177/1403494810387965 21775345

[25] Mors O Perto GP Mortensen PB. The Danish psychiatric central research register. Scand J Public Health. (2011) 39:54–7. 10.1177/1403494810395825 21775352

[26] The ICD-10 Classification of Mental and Behavioural Disorders Clinical Descriptions and Diagnostic Guidelines. World Health Organization (1992).

[27] Hjollund NH Larsen FB Andersen JH. Register-based follow-up of social benefits and other transfer payments: Accuracy and degree of completeness in a Danish inter departmental administrative database compared with a population-based survey. Scandinavian J Public Health. (2007) 35:497–502. 10.1080/14034940701271882 17852980

[28] OECD. Youth Not in Employment, Education or Training (NEET). (2017). Available online at: https://www.oecd-ilibrary.org/content/data/72d1033a-en (accessed: August 23, 2021)

[29] Birchwood M Connor C Lester H Patterson P Freemantle N Marshall M. Reducing duration of untreated psychosis: care pathways to early intervention in psychosis services. Br J Psychiatry. (2013) 203:58–64. 10.1192/bjp.bp.112.125500 23703317

[30] Edwards J Norman R Kurdyak P MacDougall AG Palaniyappan L Lau C. Unmet need for mental health services among people screened but not admitted to an early psychosis intervention program. Schizophr Res. (2019) 204:55–7. 10.1016/j.schres.2018.08.002 30121188

[31] Schmidt SJ Schultze-Lutter F Schimmelmann BG Maric NP Salokangas RK Riecher-Rossler A. EPA guidance on the early intervention in clinical high risk states of psychoses. Eur Psychiatry. (2015) 30:388–404. 10.1016/j.eurpsy.2015.01.013 25749390

[32] Ferrara M Guloksuz S Mathis WS Li F Lin IH Syed S. First help-seeking attempt before and after psychosis onset: measures of delay and aversive pathways to care. Soc Psychiatry Psychiatr Epidemiol. (2021) 56:1359–69. 10.1007/s00127-021-02090-0 33948678 PMC8319102

[33] OECD. Youth Not in Employment, Education or Training (NEET) [Internet] (2018). (accessed: July 20, 2021)

[34] Kvig EI Brinchmann B Moe C Nilssen S Larsen TK Sorgaard K. Lanthanic presentation" in first-episode psychosis predicts long service delay: the challenge of detecting masked psychosis. Psychopathology. (2017) 50:282–9. 10.1159/000478989 28797004

[35] Fusar-Poli P Salazar de Pablo G Correll CU Meyer-Lindenberg A Millan MJ Borgwardt S. Prevention of psychosis: advances in detection, prognosis, and intervention. JAMA Psychiatry. (2020). 10.1001/jamapsychiatry.2019.4779 32159746

